# Reliability of Ashworth and Modified Ashworth Scales in Children with Spastic Cerebral Palsy

**DOI:** 10.1186/1471-2474-9-44

**Published:** 2008-04-10

**Authors:** Akmer Mutlu, Ayse Livanelioglu, Mintaze Kerem Gunel

**Affiliations:** 1 Hacettepe University, Faculty of Health Sciences, Department of Physical Therapy and Rehabilitation, 06100, Samanpazari, Ankara, Turkey

## Abstract

**Background:**

Measurement of spasticity is a difficult and unresolved problem, partly due to its complexity and the fact that there are many factors involved. In the assessment of spasticity in the pediatric disabled population, methods that are easily used in practice are ordinal scales that still lack reliability. A prospective cross-sectional observational study was planned to determine the reliability of the Ashworth Scale (AS) and the Modified Ashworth Scale (MAS) in children with spastic cerebral palsy (CP).

**Methods:**

The study included 38 children with spastic diplegic CP. The mean age for the children was 52.9 months (SD: 19.6) ranging from 18 to 108 months. The functional levels of children were classified according to the Gross Motor Function Classification System. 20 children were in Level II (52.6%), 18 were in Level III (47.4%) and 9 were in Level I (23.7%). Spasticity in hip flexors, adductors, internal rotators, hamstrings, gastrocnemius were assessed by AS and MAS. Each child was assessed by three physiotherapists in two different sessions, a week apart. The intrarater reliability was determined by paired comparison of measurements for each therapist for the two assessments. Interrater reliability was determined by paired comparisons of the three therapists' measurements on the same day. The inter and intrarater reliability of the scales were evaluated by the intraclass correlation coefficient (ICC).

**Results:**

According to ICC scores, interrater reliability of AS and MAS varied from moderate to good. ICC scores of AS were between 0.54 and 0.78 and MAS were between 0.61–0.87. Test-retest results of AS and MAS varied from poor to good. ICC values were between 0.31 and 0.82 for AS and between 0.36 and 0.83 for MAS.

**Conclusion:**

The interrater and intrarater reliability of AS and MAS are related to muscle and joint characters. The repetition of measurements by the same physiotherapist, and experience may not affect reliability. These scales are not very reliable and assessments of spasticity using these scales should be therefore interpreted with great caution.

## Background

Spasticity is one feature of an upper motor neurone syndrome that may affect functionality, limit daily living activities and diminish quality of life in children with spastic Cerebral Palsy (CP) [1-5]. The assessment of spasticity is important in order to determine effectiveness of treatment on spasticity and to plan medical or surgery applications and also to measure the regulation of tonus, to decide on physiotherapy goals, and to encourage the children and their families.

However the measurement of spasticity is a difficult and unresolved problem, partly due to its complexity and the fact that there are many factors involved [6]. There are many different assessment methods for spasticity varying from clinical ordinal scales to complex electrical or orthotic equipments.

Electrophysiologic tests, electromyography, dynamic flexiometer, spasticity measurement system, pendulum test and isokinetic dynamometer are all fine examples from published literature although these methods are limited for clinical use. They are mostly used for research studies and it is hard to elicit cooperation in children [2,6-11]. In the assessment of spasticity, methods that are easily used in practice are; measuring the resistance of spastic muscles to quantify muscle tone such as the Ashworth Scales (AS), the Modified Ashworth Scales (MAS), the Tardieu Scale and the Modified Tardieu Scale (MTS). The Ashworth Scale and MAS measure spasticity and are applied manually to determine the resistance of muscle to passive stretching (Table I). The Tardiue and Modified Tardieu Scales are measured at 3 different velocities (V1, V2, and V3). By moving the limb at different velocities, the response to stretch can be more easily gauged since the stretch reflex responds differently to velocity. [8,9,12-19]. The AS, MAS, Tardieu and Modified Tardieu Scales are commonly used in children with CP [20,21].

The application of ordinal scales indicates that they still lack reliability and have some limitations in measuring spasticity. The scales offer qualitative and subjective information, concerning validity and reliability [9,22,23].

The AS and MAS need no equipment; they are easily and commonly used in the clinic [2,8,9,24-26]. However, these scales have some disadvantages because they are not standardized, stimulus is not well controlled, and also they have no reliability and validity for all muscle groups. They are not easily used statistically as they include numerical values [2,3,8,9,16,27].

In the study conducted by Bohannon and Smith, the reliability of AS in elbow flexors in patients with stroke was assessed and found reliable [8]. The reliability of AS ve MAS is better in the upper limb. The reliability of lower extremities has controversial results and has demonstrated low reliability in children with spastic CP in a few studies published [2,16,27]. Clapton et al. investigated the interrater and intrarater reliability of MAS in elbow flexors, hip adductors, quadriceps, hamstrings, gastrocnemius and soleus of 17 children with hypertonus. Elbow flexors and hamstrings had good ICC values of interrater reliability while poor interrater reliability in other muscles was observed. Hamstrings had good intrarater reliability while the other muscles had moderate reliability [28]. Fosang et al. investigated reliability of MAS, passive range of motion (PROM) and MTS in 16 children with CP. All measurements were repeated twice by six raters. The interrater reliability for PROM and MTS provided acceptable intra class correlation coefficient values, but the results for MAS were lower [19]. In the studies analyzing the reliability of AS, Sehgal reported that AS had a limited and low reliability. Pandyan et al. found that interrater reliability of AS should be addressed and Brashear et al found "good" inter and interrater reliability results of AS in patients with stroke [22,29,30]. Yam and Leung investigated the reliability of MAS and MTS in children with spastic CP. The intraclass correlation coefficients of both scales were low and did not reach the acceptable limit of 0.75. Caution should be used when these scales are applied [31].

AS and MAS are common to clinical practice and are frequently used. As the reliability of both scales are not definite and there are few studies on younger children, we planned to conduct this study. There is no study in the published literature investigating the reliability of AS and MAS together in younger children with CP. The purpose of our study was to assess the intra and interrater reliability of the AS and MAS, and to examine the reliability of both scales in the lower extremities in children with spastic CP.

## Methods

### Procedure

The study received ethical approval from Hacettepe University Ethics Committee and all parents of the children were informed about the study and their consent was obtained. A prospective cross-sectional observational study was conducted on the lower limbs of 38 spastic diplegic children (76 lower limbs in all) whose parents had given consent, and who had the inclusion criteria and were able to complete the study. Eight out of 38 children could not participate in the second assessment session as 3 children displayed anxiety and could not cope with measurement, 5 children were living out of the city and were not able to attend twice. Therefore the intrarater reliability was assessed in 30 children.

The study included 11 girls, 27 boys, a total of 38 children with spastic diplegic CP. The mean age for the children was 52.9 months (SD: 19.6) ranging from 18 to 108 months. The functional level of participants was classified according to the Gross Motor Function Classification System (GMFCS), 20 children with CP were in Level II (52.6%), 18 were in Level III (47.4%) and 9 were in Level I (23.7%) [32]. Level I represents the children who can walk without restrictions but have limitations in more advanced gross motor skills. Level II represents those who can walk without restrictions but have limitations walking outdoors and in the community. Level III represents those who can walk with assistive mobility devices but with limitations in walking outdoors and in the community.

Inclusion criteria for the study were; (i) Spastic diplegic type of CP; (ii) having had no orthopedic surgery, Botulinium toxin injection; (iii) having had no oral or intratheceal myorelaxant drugs; (iv) having had no severe limitations in passive range of motion at lower extremities and (v) having had no mental retardation. Each child was assessed by three physiotherapists in two different sessions a week apart. The intrarater reliability was determined by a paired comparison of the measurements for each therapist between the two assessments. The interrater reliability was determined by a paired comparison of the measurements of the three therapists on the same day.

The full time experience of the participating physiotherapists (A,B,C) was 16, 12, 3 years as well as 14, 8, 3 years in pediatric rehabilitation respectively. All of the measurements were taken in the supine position, the head position was in midline and the resting limb position was neutral except the hip external rotation measurement, taken in the sitting position. The scores for AS and MAS were determined according to the level of resistance during the passive movement of the antagonist muscles [8,9,23]. The muscle groups tested were hip flexors, adductors (knee extended), internal rotators of hip, hamstrings and plantar flexors (knee extended), (Table 1).

**Table 1:** Descriptions of Ashworth and Modified Ashworth Scales

***Ashworth Scale***
0 No increase in tone
1 Slight increase in tone giving catch when the limb is moved in flexion and extension
2 More marked increase in tone, but limb is easily flexed
3 Considerable increases in tone, passive movement difficult
4 Limb rigid in flexion or extension [17].
***Modified Ashworth Scale***
0 No increase in muscle tone
1 Slight increase in muscle tone, manifested by a catch and release or by minimal resistance at the end of the range of motion when the affected part(s) is(are) moved in flexion or extension
1+ Slight increase in muscle tone, manifested by a catch followed by minimal resistance through the remainder of the range of motion but the affected part(s) is(are) easily moved.
2 More marked increase in muscle tone through most of the range of movement, but the affected part(s) is easily moved.
3 Considerable increases in muscle tone, passive movement difficult
4 Affected part(s) is (are) rigid in flexion or extension

A pilot study was performed to reach an agreement among the physiotherapists about the scoring of AS and MAS, the positioning of the patient and also for agreement on speed of movement, number of repetitions of movement per joint, and the order of testing for the muscles in the lower extremities. One repetition was done per joint. The three physiotherapists agreed on an optimum speed. Assessments were performed by the three physiotherapists (A, B, C) in the same order, in a quiet room when the participants were calm and relaxed. The order of testing for the muscles were as follows: hip flexors, adductors, internal rotators, hamstrings and plantar flexors. The physiotherapists tried to perform the assessments without causing any discomfort. Each physiotherapist was assisted by the same fourth physiotherapist who did not perform any measurement and only helped maintain the positions of the subjects and recorded the scores. Assessments were performed and measured only once in the same session due to the nature of spasticity and a 30-minute interval period between the assessments was added in order to eliminate stretch reflexes occurring in the previous measurement and not to affect the following measurements. The interval period between two assessment sessions was 7 days in order not to keep the initial records in mind. Scores from the right and left sides of the body were combined for the same muscle and data from all raters were collected. Participants were assessed by using AS and MAS [8,9].

### Statistical analysis

We handled each lower extremity of the child as a seperate case and therefore different results of the right and left leg of a child did not affect each other. The intraclass correlations coefficient (ICC) was used to assess the intra and interrater reliability of AS and MAS. Fleiss and Cohen suggest that ICC is the mathematical equivalent of the weighted Kappa for ordinal data, but it can also assess reliability for more than two raters at a time and for different numbers of raters for each subject [33]. The ICC can be used for ordinal data with equal distance between intervals [34]. MAS and AS scores were considered ordinal and a value of 1.5 for MAS was assigned to ratings of 1+ to maintain equal intervals [22]. The 95% confidence interval (CI) was used to determine the statistical significance. The clinical significance was defined as poor for an ICC below 0.50, moderate for 0.50 to 0.75, and good for 0.75 or higher [34]. The software used for all calculations was SPSS 11.01 for Windows.

## Results

The AS and MAS scores of the mean value, the minimum and maximum values of AS and MAS are presented in Table 2.

**Table 2:** Distribution of Results of Ashworth Scale and Modified Ashworth Scale

			**ASWORTH SCALE**		**MODIFIED ASHWORTH SCALE**	
**Muscles**	Measurement	PT	Mean Value	Min-Max	Mean Value	Min-Max

Hip Flexors	First	A	1	0–3	2	0–4
		B	1	0–3	1	0–4
		C	1	0–2	1	0–3

	Second	A	1	0–3	2	0–4
		B	1	1–2	1	1–2
		C	1	0–2	1	0–3

Hip	First	A	2	0–3	2	0–4
Adductors (Knee extended)		B	1	1–2	2	1–3
		C	1	1–2	2	1–3

	Second	A	2	0–2	2	0–3
		B	1	1–2	2	1–3
		C	1	1–3	2	1–4

Hip Internal Rotators	First	A	1	0–2	2	0–3
		B	1	0–2	1	0–3
		C	1	0–2	2	0–3

	Second	A	1	0–2	1	0–3
		B	1	0–2	1	0–3
		C	1	0–2	2	0–3

Hamstrings	First	A	2	1–3	3	1–4
		B	2	1–3	3	1–4
		C	2	1–3	2	1–4

	Second	A	2	1–3	3	1–4
		B	2	1–3	3	2–4
		C	2	1–3	3	1–4

Plantar Flexors (Knee extended)	First	A	2	1–3	3	1–4
		B	2	1–3	3	1–4
		C	2	0–3	2	0–4

	Second	A	2	1–3	3	1–4
		B	2	1–2	3	1–4
		C	2	1–3	3	1–4

min: minimum maks: maximum

### Interrater Reliability of AS

ICC scores of AS results demonstrated good reliability for the first and second measures of hip internal rotators (ICC: 0.80, 0.78), the first measure of hamstrings (ICC: 0.78) and only the second measure of hip flexors (ICC: 0.76). Moderate reliability was found for the first and second measures of hip adductors with knee extended (ICC: 0.68, 0.72) and plantar flexors with knee extended (ICC: 0.57, 0.54), the first measure of hip flexors (ICC: 0.70) and only for the second measure of hamstrings (ICC: 0.69) (Table 3).

**Table 3:** Interrater Reliability of Ashworth and Modified Asworth Scales

**Interrater Reliability (95% Confidence Interval)**				
**Muscle**	**Measurement**	**N**	**ASHWORTH**	**MODIFIED ASWORTH**

Hip Flexors	First	76		
		76	0.70 (0.57–0.80)	0.71 (0.58–0.81)
		76		

	Second	60		
		60	0.76 (0.64–0.85)	0.74 (0.60–0.83)
		60		

Hip Adductors (Knee extended)	First	76		
		76	0.68 (0.54–0.79)	0.83 (0.75–0.88)
		76		

	Second	60		
		60	0.72 (0.57–0.82)	0.87 (0.81–0.92)
		60		

Hip Internal Rotators	First	76		
		76	0.80 (0.70–0.86)	0.84 (0.77–0.89)
		76		

	Second	60		
		60	0.78 (0.67–0.86)	0.61 (0.40–0.75)
		60		

Hamstrings	First	76		
		76	0.78 (0.68–0.85)	0.76 (0.65–0.84)
		76		

	Second	60		
		60	0.69 (0.52–0.80)	0.73 (0.59–0.83)
		60		

Plantar Flexors (Knee extended)	First	76		
		76	0.57 (0.45–0.68)	0.64 (0.52–0.74)
		76		

	Second	60		
		60	0.54 (0.39–0.67)	0.68 (0.56–0.78)
		60		

### Interrater Reliability of MAS

MAS results indicated good reliability for the first and second measures of hamstrings (ICC: 0.76, 0.73) and adductors (ICC: 0.83, 0.87), the first measure of hip internal rotators (ICC: 0.84). Moderate reliability was found for the first and second measures of hip flexors (ICC: 0.71, 0.74) and gastrocnemius (ICC: 0.64, 0.68), the second measure of hip internal rotators (ICC: 0.61) and hamstrings (ICC: 0.73) (Table 3).

### Intrarater Reliability of AS

Among three raters, the AS intrarater ICC scores were found to be ranging from poor to good (ICC: 0.31–0.82). The lowest reliability was 0.31 between the adductor measurements of rater C and the highest reliability was 0.82 between the hamstring measurements of rater C. All scores of raters are demonstrated in Table 4.

**Table 4:** Intrarater Reliability of Ashworth Scale

**Intrarater Reliability (95% Confidence Interval) N= 60**			
**Variable tested**	**A**	**B**	**C**

Hip Flexors	0.61 (0.42–0.74)	0.44 (0.21–0.62)	0.58 (0.38–0.72)
Hip Adductors (Knee extended)	0.73 (0.59–0.83)	0.63 (0.46–0.76)	0.31 (0.06–0.52)
Hip Internal Rotators	0.60 (0.42–0.74)	0.38 (0.15–0.58)	0.59 (0.39–0.73)
Hamstrings	0.49 (0.27–0.66)	0.67 (0.50–0.78)	0.82 (0.72–0.89)
Gastrocnemius	0.47 (0.25–0.64)	0.42 (0.18–0.60)	0.43 (0.20–0.62)

### Intrarater Reliability of MAS

The scores were poor to and good (ICC: 0.36–0.83). The lowest reliability was 0.36 between the hip internal rotator measurements of rater A and the highest reliability was 0.83 between the hip flexor measurements of rater C. The intrarater ICC scores of MAS are demonstrated in Table 5.

**Table 5:** Intrarater Reliability of Modified Ashworth Scale

**Intrarater Reliability (95% Confidence Interval) N= 60**			
**Variable tested**	**A**	**B**	**C**

Hip Flexors	0.74 (0.61–0.84)	0.43 (0.20–0.61)	0.83 (0.74–0.89)
Hip Adductors (Knee extended)	0.74 (0.60–0.83)	0.62 (0.43–0.75)	0.78 (0.65–0.86)
Hip Internal Rotators	0.36 (0.12–0.56)	0.41 (0.18–0.60)	0.76 (0.63–0.85)
Hamstrings	0.56 (0.36–0.71)	0.54 (0.33–0.69)	0.69 (0.54–0.80)
Gastrocnemius	0.56 (0.36–0.71)	0.70 (0.54–0.80)	0.68 (0.51–0.79)

## Discussion

In the assessment of spasticity in children with spastic CP, a number of ordinal scales such as AS, MAS and Tardieu and MTS are commonly used [20,31,35]. There is no study in the published literature investigating the reliability of AS and MAS together in younger children with CP, therefore we undertook this study. To our knowledge, this is the first study investigating the intra and interrater reliability of AS and MAS in children with spastic CP. AS and MAS measure resistance to passive movement and therefore measure hypertonia [36].

In this study, reliability in hip flexors, adductors, internal rotators, hamstrings and gastrocnemius muscle groups in children with spastic CP were investigated. The interrater reliability scores of both AS and MAS were ranged from moderate to good and the intrarater reliability scores ranged considerably from poor to good.

Various factors may affect the measurement results of reliability. While investigating the reliability of scales, related joints, anatomic and biomechanical characteristics of muscle groups as well as interrater and intrarater change and biological change should be taken into consideration [37]. Priebe et al determined that low reliability results of ordinal scales are related to problems which occur during the measurement of spasticity as well as the environment and general condition of the patient [17].

In order to eliminate these negative factors in our study, an appropriate environment regulation, the comfort of the children, the relaxation of the children, and interval periods between measurements were provided. Besides, due to its nature, spasticity is sensitive to passive stretching and velocity may affect clinical features. As passive stretching is considered to affect the following measurement results, measurements were repeated once on two different days of the study. To minimize the disadvantage of the stretching of the spastic muscle, fast stretching was avoided. The measurement criteria were standardized by a pilot study previously. The physiotherapists performed measurements in the same order and gave breaks between the measurement of the testers in order to avoid the effect of stretching.

In our study, the ICC scores of interrater reliability ranged from 0.54 to 0.80 and the intrarater reliability from 0.31 to 0.82, the gastrocnemius muscle had the lowest value in AS, and the interrater reliability of MAS was between 0.64–0.87, while the intrarater reliability was between 0.41–0.83. It may be that there is a relation to AS and MAS. We were not surprised to see that the inter reliability was higher than the intratester reliability. This confirms that these scales should be interpreted with great caution and indicates that even the same rater has the possibility of making an error. The repetition of measurements by the same physiotherapist, and experience may not affect reliability as we mentioned in the conclusion of our study.

Although the interrater reliability of AS and MAS were similar in our study, the intrarater reliability of MAS had higher scores than the intrarater reliability of AS. This result may arise from the common use of MAS in practice by raters who had experience in pediatric physiotherapy. Fosang stated that MAS had better intrarater reliability compared to interrater reliability and it should only be used by a single rater for the same participant rather than different raters [19]. The interrater reliability was higher than the intrarater reliability of our MAS results when compared to the results of the study conducted by Fosang and Clapton. This may be due to the low number of raters in our study [19,28].

The mean value of MAS was 0.87 for the intertester reliability of adductor muscles and 0.68 for the intertester reliability of plantar flexors. Yam and Leung investigated the interrater reliability of MAS and MTS for hip adductors and plantar flexors in children with spastic CP. Their results showed that the intraclass correlation coefficients of both scales were low and did not reach the acceptable limit of 0.75. We had similar results in the plantar flexors although they were different in the adductors.

Our result for the adductor muscles may be related to the laws of physics. Power and load arm of these muscle groups are longer compared to those of the plantar flexors. In addition, the range of motion of the adductor muscle groups is greater than that of the plantar flexors. These may provide a higher reliability of the adductor muscles.

There are few studies examining the reliability of AS and MAS in one single study, however recent studies have focused on the reliability of MAS [19,28].

There have been studies focusing on the reliability of AS and MAS on the adult population. Haas et al used AS and MAS for assessing lower extremity spasticity in 33 adult paraplegic patients and found AS to be more reliable than MAS [38]. Ansari et al assessed wrist spasticity by AS and MAS in patients with stroke and reported no difference for the interrater reliability between AS and MAS [39]. Reliability scales are also affected from the assessed muscles and personal characteristics of subjects. Ease in manipulation as well as supporting the lower extremities in children and the range of motion capability due to the muscle group which is assessed are the probable characteristics mentioned above [8,22,28]. Also, our sample group consisted of children who are younger than those of most sample groups with CP. Therefore, this could have affected our results. Younger kids would be easier to move due to smaller limbs (especially for the proximal muscles which are addressed briefly) but would be harder to test due to reasons of adherence since they are so young.

## Conclusion

Nevertheless, recent studies on this issue may guide future studies. The interrater and intrarater reliability of AS and MAS are related to muscle and joint characters. The repetition of measurements by the same physiotherapist, and experience may not affect reliability. These scales are not very reliable and assessments of spasticity using these scales should be therefore interpreted with great caution. Future research studies are required to analyze factors affecting reliability in children with CP.

## Conflict of interest

The author(s) declare that they have no competing interests.

## Authors' contributions

AM carried out study design, data collection, statistical analysis, data interpretation, manuscript preparation, literature search. AL participated study design, data collection, statistical analysis, data interpretation, manuscript preparation, MKG participated study design, data collection, statistical analysis, manuscript preparation, literature search.

## Pre-publication history

The pre-publication history for this paper can be accessed here:


